# Supplementary figures and images for: Monitoring plasma nucleosome concentrations to measure disease response and progression in dogs with hematopoietic malignancies
Source: PLoS One. 2023 May 10;18(5):e0281796. doi: 10.1371/journal.pone.0281796 (PMC10171669; doi:10.1371/journal.pone.0281796)

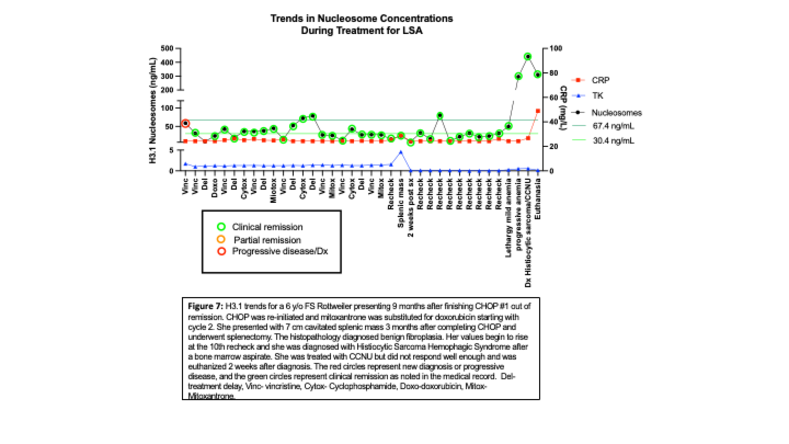

Supplement: S1 Fig — CHOP was re-initiated and mitoxantrone was substituted for doxorubicin starting with cycle 2. She presented with 7 cm cavitated splenic mass 3 months after completing CHOP and underwent splenectomy. The histopathology diagnosed benign fibroplasia. Her values begin to rise at the 10th recheck and she was diagnosed with Histiocytic Sarcoma Hemophagic Syndrome after a bone marrow aspirate. She was treated with CCNU but did not respond well enough and was euthanized 2 weeks after diagnosis. The red circles represent new diagnosis or progressive disease, and the green circles represent clinical remission as noted in the medical record. Del- treatment delay, Vinc- vincristine, Cytox- Cyclophosphamide, Doxo-doxorubicin, Mitox- Mitoxantrone. (TIF) [file pone.0281796.s001.tif]

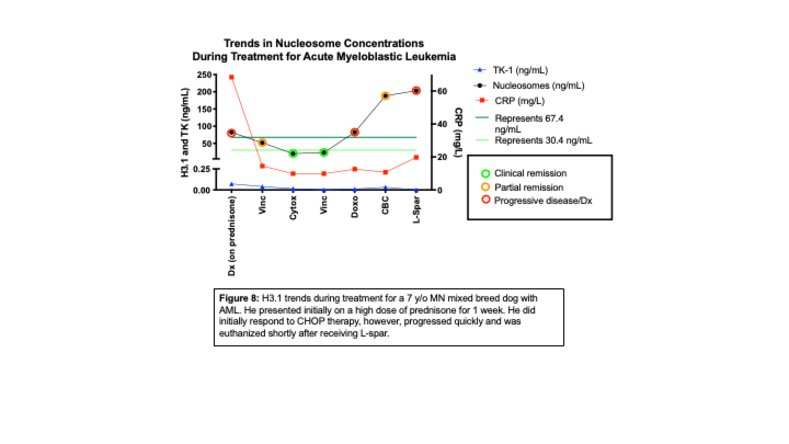

Supplement: S2 Fig — He presented initially on a high dose of prednisone for 1 week. He did initially respond to CHOP therapy, however, progressed quickly and was euthanized shortly after receiving L-spar. (TIF) [file pone.0281796.s002.tif]

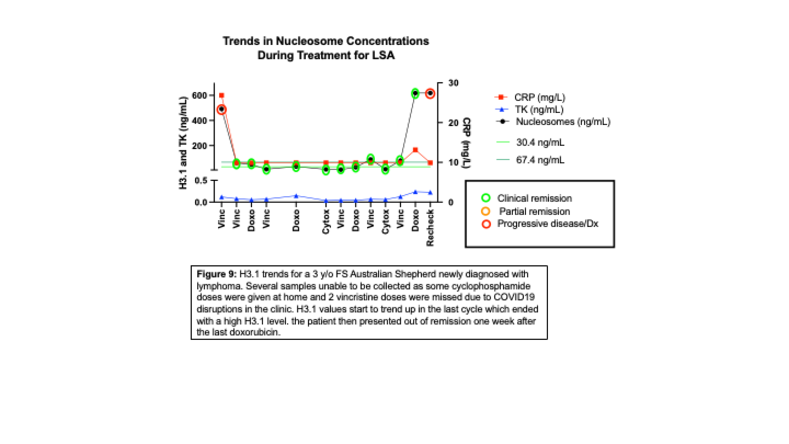

Supplement: S3 Fig — Several samples unable to be collected as some cyclophosphamide doses were given at home and 2 vincristine doses were missed due to COVID19 disruptions in the clinic. H3.1 values start to trend up in the last cycle which ended with a high. (TIF) [file pone.0281796.s003.tif]
