# Supplementary material for: Monitoring plasma nucleosome concentrations to measure disease response and progression in dogs with hematopoietic malignancies
Source: PLoS One. 2023 May 10;18(5):e0281796. doi: 10.1371/journal.pone.0281796 (PMC10171669; doi:10.1371/journal.pone.0281796)
Supplement: Attachment — Submitted filename: Response to reviewers r2- longitudinal paper.docx [file pone.0281796.s005.docx]

Response to reviewers- revision 2

6. Review Comments to the Author

Please use the space provided to explain your answers to the questions above. You may also include additional comments for the author, including concerns about dual publication, research ethics, or publication ethics. (Please upload your review as an attachment if it exceeds 20,000 characters)

Reviewer #1: (No Response)

Reviewer #2: Thank you for your thorough responses to my previous comments. Only a few areas of clarification remain necessary as listed below.

Methods:
Add a brief statement in the methods section about what results for the Ni.Q assay were considered normal, grey zone, and elevated and/or reference figure 1 for interpretation of results.

*This has been added in lines 166-169.*

Results:
Lines 318-323: I assume nucleosome concentrations between dogs with CR vs PR vs SD are not statistically different. Please state that result here.

*This has been added to lines 323-325.*

Table 3:
With the added data provided in lines 318-323, it is confusing to compare with the data in table 3. I see that the median nucleosome concentrations at SD and PR are 25.7 and 21.8, respectively, but table 3 shows SD and PR dogs with a mean of 50.51. Ideally there would be more consistency in reporting of means vs medians depending on whether data is normal or not.

*Thank you for catching this error. This has been corrected to 23.85 in table 5.*

Lines 362-364: Please state the statistical analysis you ran to come to this conclusion. Readers want to know which of the groups are significantly different.

*This information has been added to the last paragraph in the methods section.*
